# A Molecular Docking Study Reveals That Short Peptides Induce Conformational Changes in the Structure of Human Tubulin Isotypes αβI, αβII, αβIII and αβIV

**DOI:** 10.3390/jfb14030135

**Published:** 2023-02-28

**Authors:** Oluwakemi Ebenezer, Nkululeko Damoyi, Michael Shapi, Gane Ka-Shu Wong, Jack A. Tuszynski

**Affiliations:** 1 Department of Chemistry, Faculty of Natural Science, Mangosuthu University of Technology, Umlazi 4031, South Africa; 2 Department of Biological Sciences, University of Alberta, Edmonton, AB T6G 2E9, Canada; 3 Li Ka Shing Institute of Virology, University of Alberta, Edmonton, AB T6G 2E1, Canada; 4 Department of Physics, University of Alberta, Edmonton, AB T6G 2E1, Canada; 5 Department of Oncology, Cross Cancer Institute, University of Alberta, Edmonton, AB T6G 1Z2, Canada; 6 Department of Mechanical and Aerospace Engineering, (DIMEAS), Politecnico di Torino, 10129 Turin, Italy

**Keywords:** antimicrobial peptides, anticancer, PATCHDOCK, FIREDOCK, ClusPro

## Abstract

Microtubules are cylindrical protein polymers assembled in the cytoplasm of all eukaryotic cells by polymerization of aβ tubulin dimers, which are involved in cell division, migration, signaling, and intracellular traffic. These functions make them essential in the proliferation of cancerous cells and metastases. Tubulin has been the molecular target of many anticancer drugs because of its crucial role in the cell proliferation process. By developing drug resistance, tumor cells severely limit the successful outcomes of cancer chemotherapy. Hence, overcoming drug resistance motivates the design of new anticancer therapeutics. Here, we retrieve short peptides obtained from the data repository of antimicrobial peptides (DRAMP) and report on the computational screening of their predicted tertiary structures for the ability to inhibit tubulin polymerization using multiple combinatorial docking programs, namely PATCHDOCK, FIREDOCK, and ClusPro. The interaction visualizations show that all the best peptides from the docking analysis bind to the interface residues of the tubulin isoforms αβl, αβll, αβlll, and αβlV, respectively. The docking studies were further confirmed by a molecular dynamics simulation, in which the computed root-mean-square deviation (RMSD), and root-mean-square fluctuation (RMSF), verified the stable nature of the peptide–tubulin complexes. Physiochemical toxicity and allergenicity studies were also performed. This present study suggests that these identified anticancer peptide molecules might destabilize the tubulin polymerization process and hence can be suitable candidates for novel drug development. It is concluded that wet-lab experiments are needed to validate these findings.

## 1. Introduction

Microtubules are cylindrical protein polymers assembled in the cytoplasm of all eukaryotic cells by polymerizing αß-tubulin heterodimers, which are involved in multiple biological functions such as cell division, migration, signaling, and intracellular traffic. These functions, especially the formation of mitotic spindles, make them important participants in the initiation and proliferation of cancerous cells and their subsequent metastases [1,2]. They exhibit an irregular temporal pattern of assembly and disassembly, which has been termed dynamic instability [3]. In addition, altered tubulin polymerization is detrimental to the formation of the tumor vascular system during the process of angiogenesis, which is a hallmark of cancer. Hence, unsurprisingly, the microtubular cytoskeleton and its building blocks, tubulin dimers, have become one of the key targets in cancer chemotherapy [4]. It is important to note that both α- and β-tubulin monomers are expressed by different genes and occur in the form of numerous isotypes, which differ in their amino acid sequence leading to slight structural changes, especially in the binding pockets of tubulin-targeting ligands [5]. Moreover, different β-tubulin isotypes exhibit a unique dynamic behavior manifested by different interactions with ligands, such as anti-tubulin drugs, both in vitro and in mammalian cells [6,7,8]. A promising tactic to disrupt tubulin polymerization and the development of microtubules is to interfere with the protein–protein interactions responsible for the self-assembly of microtubules.

While the traditional approach to cancer chemotherapy has been based on small molecule development, short peptides have recently become the focus of new efforts to find more efficacious and less toxic drug candidates. Extensive research has been undertaken to investigate the use of peptides with a good safety profile to inhibit cancer and many other diseases [9,10]. Pieraccini et al. [11] reported modeling protein-protein interfaces and identified the amino acids responsible for peptide–tubulin binding. Thus, engineered peptides that produce anti-tubulin activity both in vitro and in cultured cells have been discovered [11]. This is of major significance since small molecules typically bind to pockets and grooves on target proteins but are seldom suitable for binding to flat protein-protein interfaces. Tubulin polymerization has been targeted by natural peptides or depsipeptides isolated from a wide range of organisms but with atypical amino acids. Among the peptides, dolastatin or cryptophycins, are regarded as promising anticancer drugs. At the same time, derivatives of these peptides such as dolastatin 10, crytophycin 1, phomopsin A, and hemiasterlin impede the binding of vinca alkaloids to tubulin in an uncompetitive way. Therefore, the subject of intensive pharmacological research is to identify similar pharmacophores. In addition, isotypes of tubulin βII, βIII, and βIV were also found to have a degree of difference in chemical affinities for various anticancer drugs such as taxol, colchicine, DAMA-colchicine, and nocodazole [12,13,14]. As a result, these drug-resistant tubulin isotypes have become attractive targets for developing novel anticancer agents. While treatments based on targeting microtubules have transformed cancer therapy, drug resistance and detrimental side effects that accompany the action of all known tubulin-binding agents remain significant drawbacks. It is therefore increasingly advisable to adopt new strategies by exploring the different profiles of tubulin isotypes in an effort to overcome the drawbacks plaguing the existing tubulin-binding drugs. Herein, we utilized peptide structure prediction, homology modeling, molecular docking, molecular dynamics simulations, and binding free-energy calculations to identify human tubulin isotypes-targeting peptides from a set of short antimicrobial peptides with potential for anticancer repurposing. We hope that the series of peptides identified in this study may offer benefits for the early discovery of a pharmacophore of interest because of their promising binding interactions with the family of tubulin protein isoforms predicted computationally in our work.

## 2. Material and Methods

### 2.1. Peptide Screening and Preparation

We explored the DRAMP (data repository of antimicrobial peptides) database in this work [15]. DRAMP is an open-access and manually curated database containing diverse annotations of AMP, including information on their sequences, structures, biological activities, physicochemical, patent, clinical, and references of the peptides. DRAMP currently contains 22,407 entries, 6032 of which are general AMPs (having both natural and synthetic AMPs), 16,110 patented AMPs, 77 AMPs in drug development (preclinical or clinical stage), and 188 stapled antimicrobial peptides belonging to specific AMPs. To expand the scope of AMPs’ design, DRAMP also holds 5909 candidate AMPs screened by some platforms whose antibacterial activities have not been assayed yet. In this study, we screened 826 peptides derived from plant sources. Anticancer peptides were extracted from the database. The peptides that did not have accurate information regarding their activity or function in the database were discarded. The peptides with more than 50 amino acids in length were also removed as being unlikely to hold promise for drug development. In addition, anticancer peptides with nonentity amino acids in their sequences were eliminated. The PEP-FOLD 2 webserver was used further to predict the information structure of 13 sequence peptides [16]. This web server visualizes the peptide structure using a hidden Markov model suboptimal sampling algorithm [17]. It uses a coarse-grained energy force field without including conformational entropy.

### 2.2. Homology Modeling and Protein Preparation

The sequences of human α and β tubulin isotypes were downloaded from the universal protein resource (UniProt) [18] (Q71U36: α-tubulin, P02773, Q13885, Q13509 and, P04350: βl-βlV-tubulin proteins). The 3D structure of bovine tubulin (Q2HJ86 and Q6B856: αβ-tubulin chain (*Bos taurus*)) was downloaded from the Protein Data Bank (PDB ID: 1SA0) as the template structure. The complex includes two tubulin αβ heterodimers, while the native ligand, namely, colchicine, is bound to β subunits at the interface with the α subunit. The homology model of the human tubulin was built with SWISS-MODEL [19]. The native ligand in the template structure was deleted before homology modeling was performed. The stereochemical quality of the template (referred to as tubulin 1SA0 hereafter) and the various tubulin isotypes were evaluated using PROCHECK [18]. Subsequently, the Verify-3D [20] and ERRAT [21] algorithms were employed to check the reliability of the generated models. The Ramachandran plots for all the isotypes are shown in Figure S1. All the structures resulting from the modeling αβl, αβll, αβlll, and αβlV were further subjected to protein preprocessing, optimization, and minimization using the protein preparation in the Schrodinger suite [22].

### 2.3. Molecular Docking

After cleaning the proteins using the protein preparation tool in the Schrodinger software suite, the output was used for the protein-peptide molecular docking. PATCHDOCK [23] and ClusPro [24] web servers were used to analyze their interactions. FIREDOCK (Fast Interaction Refinement) webserver was used for further refinement [25]. The top 10 model solutions were determined and analyzed. The PATCHDOCK web server with default parameters was used to predict the best conformations. Since a rigid approach was utilized to obtain the docking solutions and because during protein−protein interactions, both side chains and backbones might change their conformation. The top 10 solutions were subjected to the FIREDOCK webserver to refine the interaction of protein–protein complexes resulting from molecular docking [25]. FIREDOCK performs side chain optimization and rigid body minimization to provide more extensive refinement. Subsequently, scoring and ranking identified the near-native advanced solutions. The final selection of the best-docked complexes was based on the global energy of the bound, predicted complexes after the refinement.

### 2.4. Molecular Dynamics

The molecular dynamics (MD) simulations were evaluated using the Desmond simulation package embedded in the Schroedinger suite [26]. Before the MD simulations were performed, the molecular system, including the protein, water molecules, and ions, was built. The water molecules were described using the TIP3P (transferable intermolecular potential with 3 points) model in an orthorhombic cubic box. The boundary condition box volume was computed according to the complex type and counter ions, including Na^+^ and Cl^−^, to neutralize the system. The NPT ensemble with temperature and pressure of 300 K and 1 bar, respectively, was used in all the analyses for all the selected docked peptides in the αβl-αβlV tubulin receptor. The force field of OPLS_2005 was applied. Simulation progress was verified stepwise every 50 ps. The NPT assembly was launched following the simulation process, which covers the production of 100 ns. The frames were assembled and examined using the simulation interaction diagram that helped determine the fluctuations.

### 2.5. Post Molecular Dynamics MM-GBSA

The Molecular Mechanics-Generalized Born and Surface Area Continuum Solvation (MM/GBSA) was calculated for each MD trajectory using the thermal MM/GBSA script [27]. This was run via the Python command line. The average binding energy was calculated for 20 snapshots from the overall trajectory of 100 ns.

### 2.6. Physicochemical, Allergenicity, and Toxicity Prediction

Physicochemical, allergenicity, and toxicity assessments of the best peptides from the docked analysis were evaluated. The physiochemical properties of the best peptides were evaluated using ProtParam tools [28], which allow predicting the relevant properties, such as molecular weight, net charge at pH 7, peptide properties, stability, and charge. The AllerTop server was used to evaluate the nonallergenic nature of the peptides [29].

## 3. Results and Discussion

**Figure 1:**
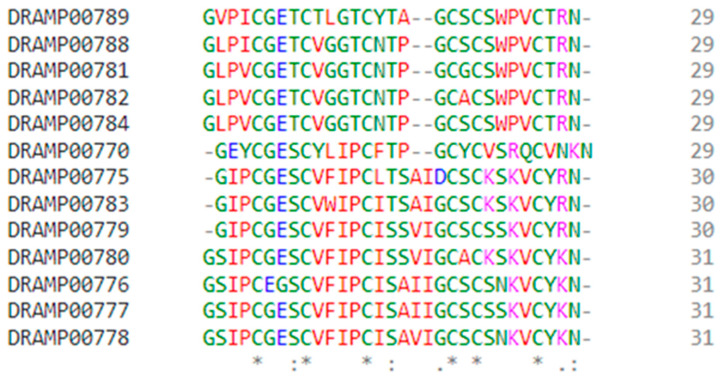
Sequenc aligment (*) denotes fully conserved residue; (:) denotes conservation between groups of significant similar properties; and (.) denotes conservation between groups of weakly similar properties.

**Figure 2:**
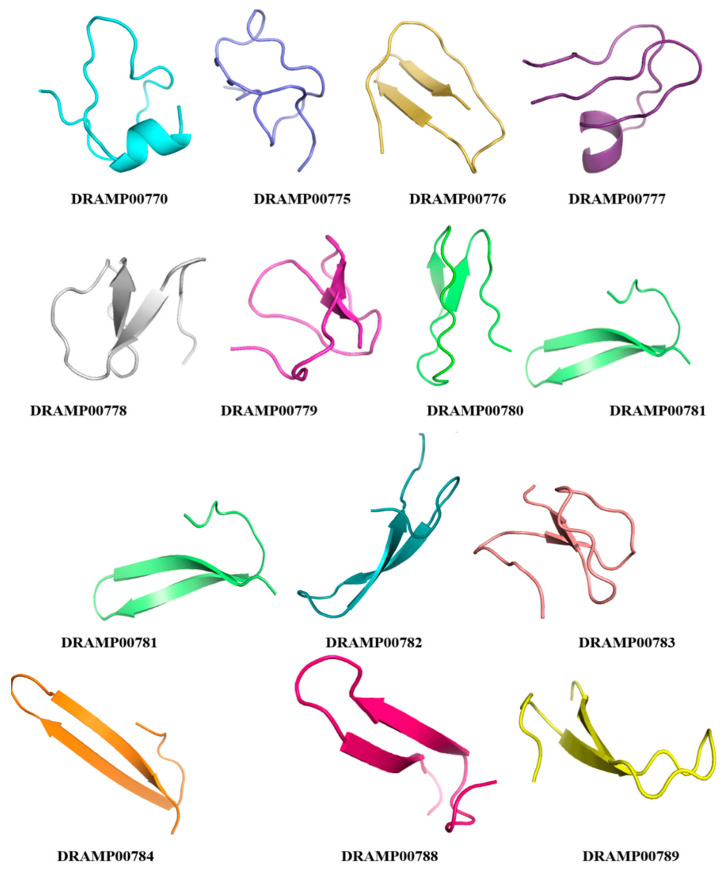
The predicted three-dimensional structures of the peptides extracted from the DRAMP database with anticancer properties using PEPFOLD 2.

We retrieved plant peptides from the DRAMP database. The 13 peptides shown in Table 1 and Figure 1 were extracted from the set of plant-based peptides. Figure 2 displays the respective structures of the peptides. Our 3D predicted structures may not represent the native conformation in the plant, but they do represent at least one version of what might be created in the wet lab experiments. Which are linear cyclotides and they are aimed to be anticancer lead compounds.

The Ramachandran plots obtained by PROCHECK found 85.1% of residues in the most favored regions, 11.4% in the additional allowed regions, and 2.3% in generously allowed regions for αβI (see Table S1 and Figure S1). For αβII, 83.4% of residues in most favored regions, 13.3% in additional allowed regions, and 1.1% in generously allowed regions were found. In the αβIII, 83.7% of residues in the most favored regions, 12.9% in additional allowed regions, and 2.4% in generously allowed regions were observed. On the other hand, in αβIV tubulin, 84.1% of residues are found in most favored regions, 12.7% in additional allowed regions, and 2.3% in generously allowed regions. The results from the overall quality factor obtained by the ERRAT agreed with the acceptable range (>50 for a high-quality model) [30]. The overall quality factor obtained by the ERRAT tool for the isotypes αβI-αβIV was found to be 91.32%, 80.86%, 82.68%, and 90.7%, respectively. Subsequently, VERIFY3D was used to confirm that at least 80%, 97.56%, 96.52%, and 97.79% of the amino acids of all the analyzed isotypes achieved an average 3D/1D score higher than 0.2. In particular, most residues are found in the most favored regions; therefore, the built model was deemed reliable.

The identification of the binding site is significant for elucidating the actual binding mechanisms as well as the interaction between a drug and a protein. To characterize this, we used molecular docking and MD simulations, which are standard, generally accepted theoretical prediction approaches for examining peptide binding sites in the various protein receptors. ClusPro was used to reexamine the peptides with the best-docked results from the FIREDOCK. The ClusPro score of docked peptides in αβIII and αβIV tubulin corroborates the result of the FIREDOCK. However, the results of αβI and αβII-tubulin obtained from CluPro and FIREDOCK do not corroborate it. Further, the peptides with the lowest global energy from the FIREDOCK (Table 2) were utilized for further analysis. The MD simulation analysis was carried out on the complexes in order to answer many relevant inquiries, such as the stability and accuracy of the binding mode and the ligand stability over the simulation period. Most of the interactions predicted in the docking analysis of the lead peptides were stable within the binding site. They interacted appropriately with different target regions by forming hydrogen bonds, as well as hydrophobic and electrostatic connections directly with protein side chains.

### 3.1. Peptide-αβI-Tubulin Complex

**Figure 3:**
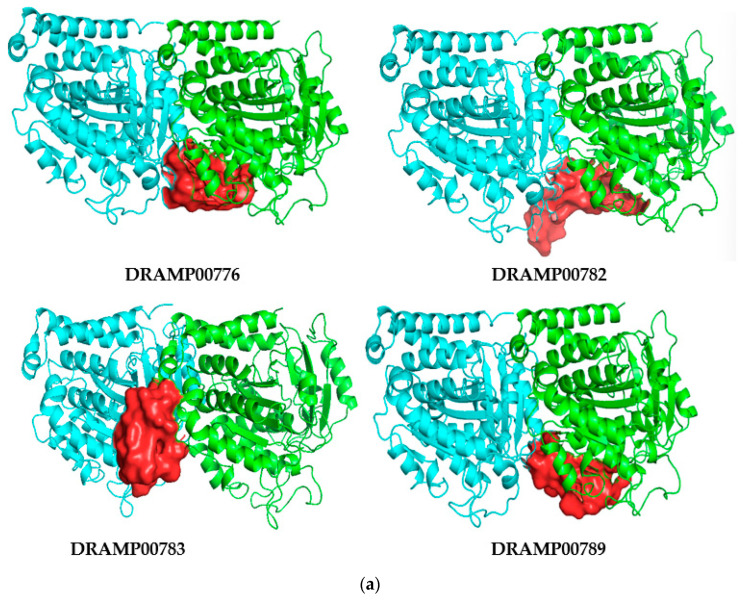

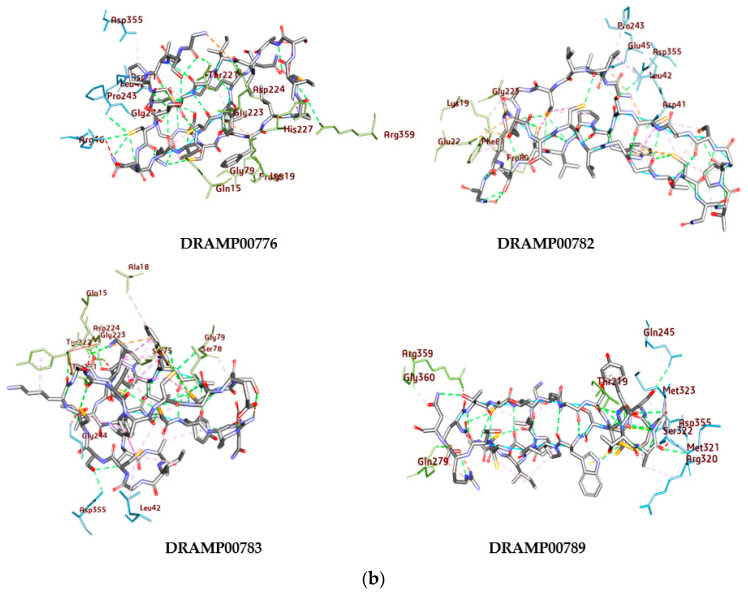
(**a**). The three-dimensional structures of DRAMP0776, DRAMP00782, DRAMP00783, and DRAM00789 (red surface) occupy the binding site of the αβI-tubulin receptor (light and flat red ribbon). (**b**). The two-dimensional structures of DRAMP0776, DRAMP00782, DRAMP00783, and DRAM00789 αβI-tubulin complexes reveal the range of the interactions and heterogeneity of the ligands’ positionings, as observed from the docking simulations. The protein receptors (green and light blue) are shown as sticks.

The DRAMP00782 peptide (Figure 3a) had the best FIREDOCK global energy estimate of −65.44 kcal/mol. The binding energies for the DRAMP0776, DRAMP00783, and DRAM00789 peptides in FIREDOCK were estimated as −65.14, −56.63, and −54.12 kcal/mol, respectively, whereas the binding energies in ClusPro were −858.2, −738.0, −761.9, and −983.3 kcal/mol, respectively. The nonbonded interactions between the peptide molecules and the αβI-tubulin receptor are shown in Figure 3b. The DRAMP00776-αβ-tubulin complex was stabilized by five hydrogen bonds at αAsp224, αThr221, αArg359, βArg46, αGly223, and βASP355 and accompanied by one hydrophobic bond at αHis227. The DRAMP0782 peptide and αβ-tubulin receptor formed seven hydrogen bonds at βAsp355, αLys19, and αGly223, accompanied by four hydrophobic interactions at βPro243, βLeu42, αPro80, and αPhe81, and four electrostatic interactions at αLys19, βAsp355, βGlu45 and αGlu22 (Figure 2b, Table 3). DRAMP00783 peptide and αβ-tubulin receptor complex formed five hydrogen bonds at αSer78, βGly79, αGlu223, αTyr222, αAsp244, βAsp355, αGln15, αSer75, αThr221, αSer78, and αAsp224 and three hydrophobic interactions at αTyr222, βLeu42, and αAla18. The DRAM00789 peptide and αβ-tubulin receptor complex displayed ten hydrogen bonds at αGln279, βMet321, βMet323, αThr219, βAsp355, βGln245, αGly360, βSer322, and βASP355 and one hydrophobic bond at βArg320 (see Figure 2b, Table 3). Peptide binding to the receptor may cause the target molecule to inhibit its interactions. All these results confirmed that the four peptides could effectively repress tubulin interactions by occupying the interface of the αβI-tubulin dimer.

**Figure 4:**
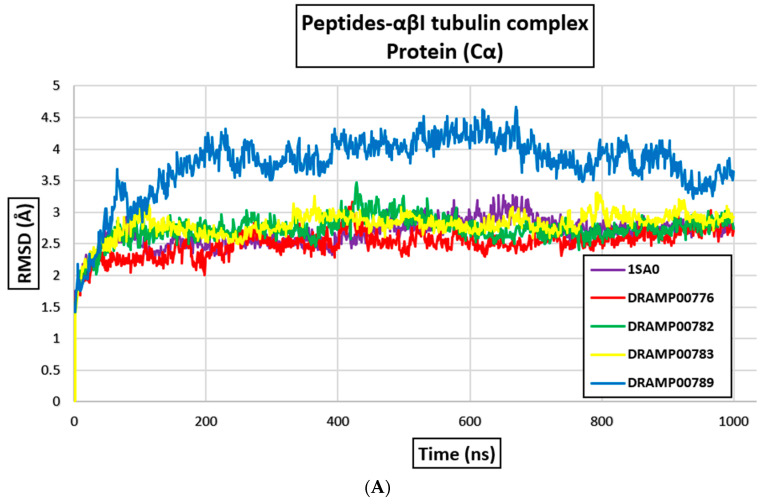

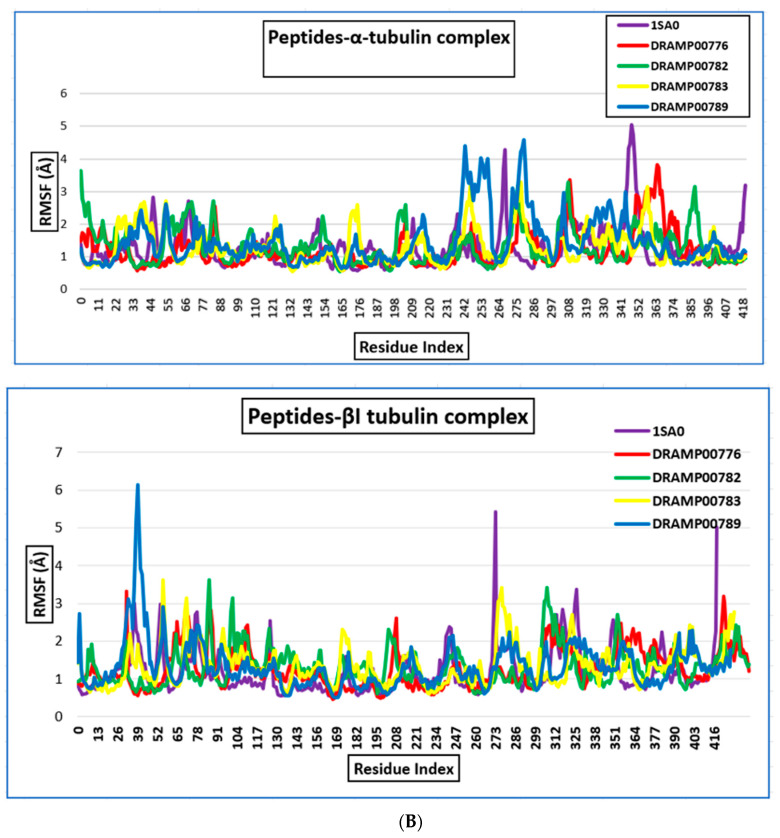
(**A**) RMSD of αβI-tubulin proteins after MD and (**B**) RMSF of α and βI-tubulin monomers after MD simulations.

The molecular dynamics simulations of the active peptides bound to the αβ tubulin heterodimer were carried out to confirm the structural rigidity and to validate the docking outcomes for the complexes. RMSD values of C-alpha atoms (Cα) have been studied to understand structural inflexibility. The results demonstrated that the DRAMP00776, DRAMP0782, DRAMP00783, and DRAM00789 complexes showed initial RMSD increases due to instability. The increase in RMSD was higher for the DRAM00789 peptide complex than for the other complexes. This result corroborates the molecular docking result. The trend in the RMSD was maintained between 40 and 65 ns, after which instability occurred. Meanwhile, DRAMP00776, DRAMP0782, and DRAMP00783 maintained stability from 70 ns to the end of the simulation period. Although the DRAMP00789 complex had a higher RMSD trend during the initial phase, the RMSD profile decreased with an increase in the simulation time (from 40 ns to 100 ns, see Figure 4). The average RMSD profile of the complexes formed with DRAMP00776, DRAMP00782, and DRAMP00783 was between 2.5 Å and 2.8 Å, respectively. The overall RMSD estimate for DRAMP00779 was 3.5 Å, and fluctuations were observed throughout the simulation time, indicating overall structural instability because higher RMSD profiles for Cα atoms are indicators of low stability (Figure 4A). DRAMP00776 formed hydrogen bonds with the residues, αLys19, αAsn226, αArg276, αGln15, αGly71, αSer75, αGly79, αPro80, αGly223, and αGlu22, respectively. Compared to the interacting residues before MD simulations, DRAMP00783 peptides shift from the initial binding position. DRAMP00783 interacts with αGln15, βAsp355, βGln245, αAsp74, and βAsp355 via hydrogen bonding, which is accompanied by three hydrophobic interactions via βMet323, βVal353, and αTyr222 (Table 2). It was also observed that the higher the hydrophobic interaction, the better the binding energy. RMSF plots provide information on the flexible regions of the MD simulated structures. Flexible regions display a higher RMSF value, while constrained regions display a low RMSF value. There were no significant fluctuations in amino acid residues in the β-monomer after binding the peptides. The calculated RMSF for DRAMP00776, DRAMP00782, and DRAMP00783 was analyzed, and the peptides exhibited lower flexibility, below the 4 Å range, as shown in Figure 4B.

### 3.2. Peptide-αβII-Tubulin Receptor Complex

**Figure 5:**
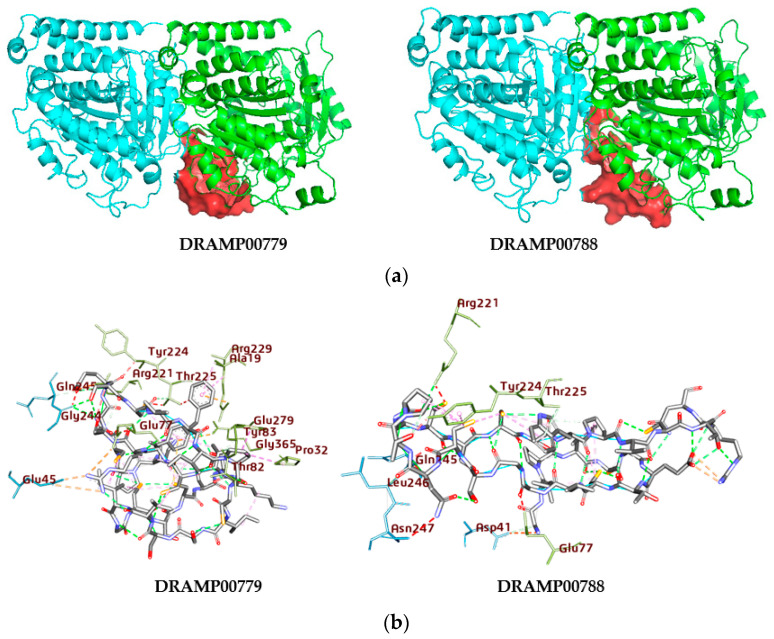
(**a**) The three-dimensional structures of DRAMP0779 and DRAMP00788 (red surface) occupying the binding site of αβII-tubulin dimer (light and red flat ribbon). (**b**) The two-dimensional structures of DRAMP0779 and DRAM00788 αβII-tubulin complexes unveil the range of the interactions and heterogeneity of the ligands’ positionings, as observed in the docking simulation. The proteins (green and light blue) are shown as sticks.

**Figure 6:**
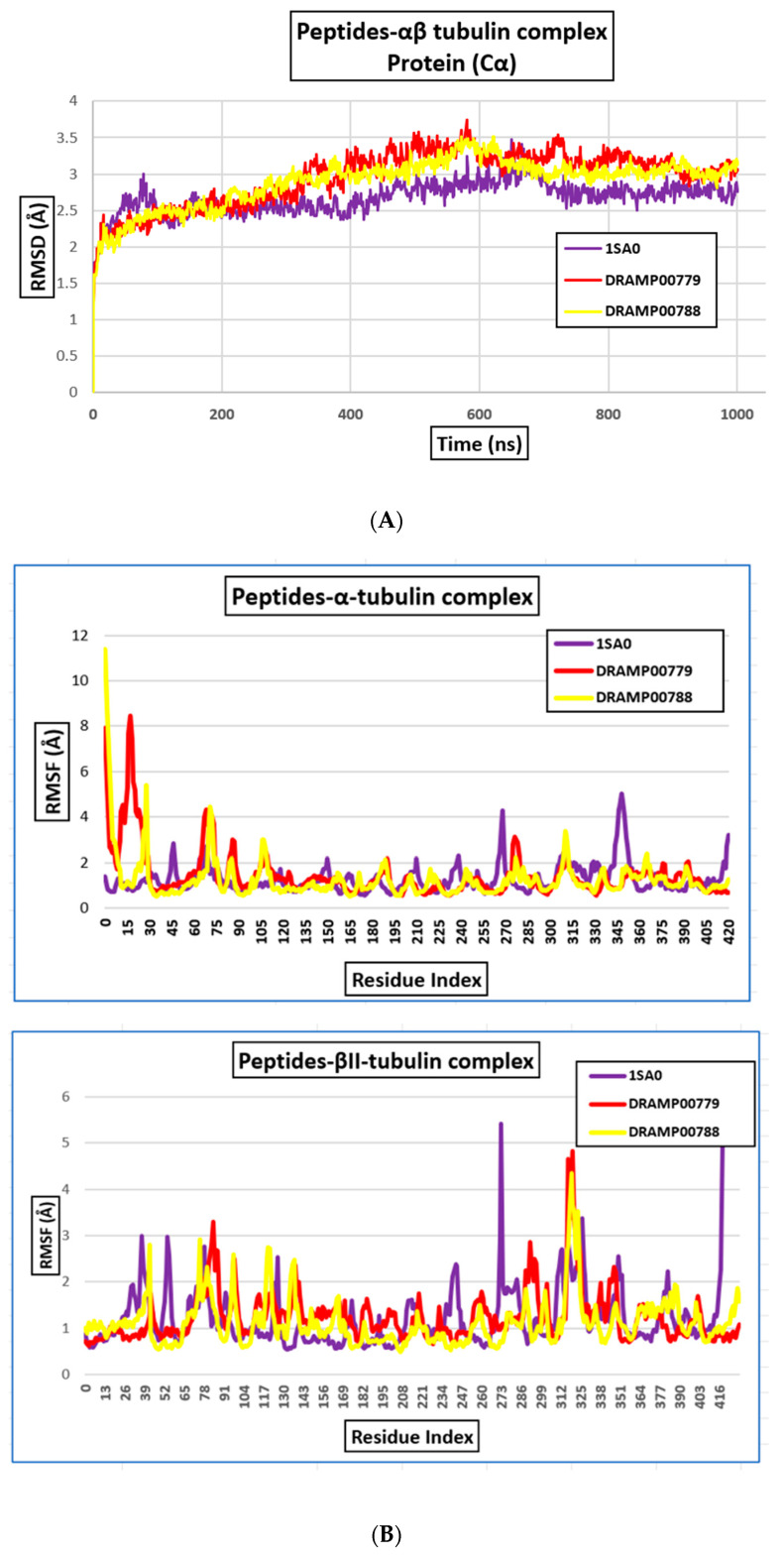
(**A**) RMSD of αβII-tubulin proteins after MD simulations and (**B**) RMSF of α and βII-tubulin proteins after MD simulations.

The binding region surrounding the peptides consists of residues within a 4 Å range from the peptide atoms (Figure 5a). Subsequently, two peptides were found to have potent binding energy, namely, DRAMP00779 and DRAMP00788, respectively, after docking the extracted peptides to the αβII-tubulin dimer. For the DRAMP00779 complex, the residues αArg221, αGlu279, αGlu77, αThr82, αArg229, αThr225, αThr82, αTyr83, αAla19, αGly365 were detailed in the α-tubulin and βGln245, βGlu45 in the β-tubulin subunit (Figure 5b). While residues αArg221, αThr225, and αTyr224 were detailed in the α-tubulin βLeu246, βAsp41, and βAsp41 in the β-tubulin subunit for the DRAMP00788 complex (Figure 5b, Table 4). The trajectories obtained from a set of MD simulations correspond to conformational changes of the complex of DRAMP00779 and DRAMP00788, and the corresponding data for individual biological systems were analyzed. The difference in the RMSD of the liganded and the unliganded tubulin heterodimer is not so dramatic (Figure 6A). The structural motions of the residues in each tubulin subunit achieve additional stability upon binding the peptides. The primary residues involved in the interaction of the DRAMP00779 complex after 100 ns of simulation time include αGly81, αArg221, βMet323, αGly81, αThr80, βAsp355, αArg221, αGly365, βMet321 (conventional and carbon–hydrogen bonds). Hydrophobic and electrostatic interactions also stabilized the complex. While DRAMP00788 interacted with residues, αAsp76, αGlu77, αArg221, αGly365, αAsp76, αGlu77, αArg221, αThr80, αGly81, βMet323, βLys324, βMet323, βAsp355, βLeu42, βMet321, and βLys324. Furthermore, the peptide formed distinct interactions such as electrostatic, hydrophobic, and hydrogen bonds with the intermediate domain residues of the αβ tubulin dimer (206–381). The domain opens with helices H6 and H7, a long loop, and helix H8 at the longitudinal interface sandwiched between the monomers. Many areas of the outer surface of tubulin are negatively charged and can attract hydrogen ions [31]. These regions play a role in tubulin–tubulin interactions and tubulin interactions with motor proteins such as kinesin. The MD results demonstrate that DRAMP00779 interacts electrostatically more strongly than DRAMP00788. To explore the atomic fluctuations at the binding sites in detail, we determined the RMSF value of each amino acid residue at the αβ tubulin binding site (Figure 6B). The residues fluctuated at the N-terminal domain (amino acids 1 to ~205). The atomic fluctuation profile shows a low fluctuation of the DRAMP00779 and DRAMP00788–tubulin complex in the α-tubulin compared to the β-tubulin monomer.

### 3.3. Peptides-αβIII-Tubulin Dimer Complex

**Figure 7:**
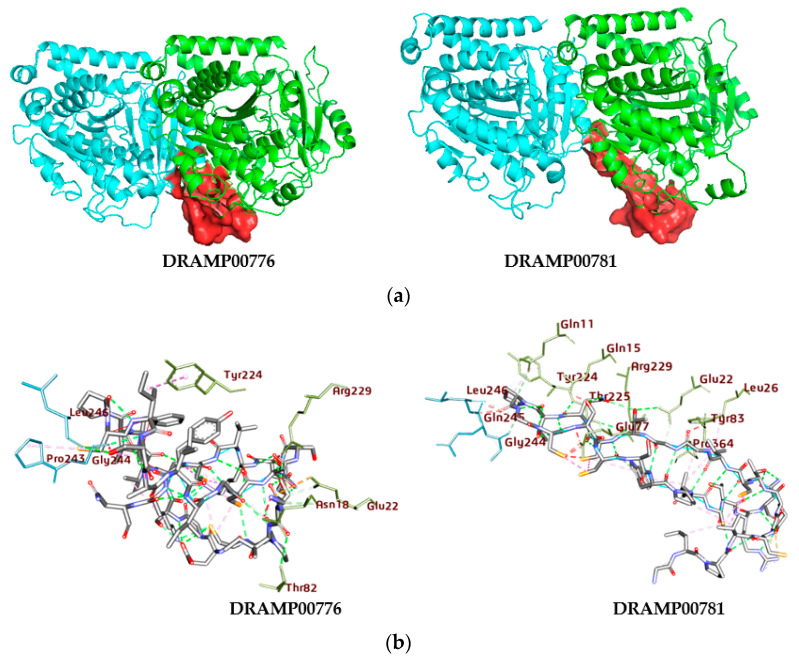
(**a**) The three-dimensional structures of DRAMP0776 and DRAMP00781 (red surface) occupy the binding site of the αβIII-tubulin dimer (light and flat red ribbon). (**b**) The two-dimensional structures of DRAMP0776 and DRAM00781 αβIII-tubulin complexes reveal the range of the interactions and heterogeneity of the ligands’ positionings, as obtained in the docking exercise. The proteins (green and light blue) are shown as sticks.

**Figure 8:**
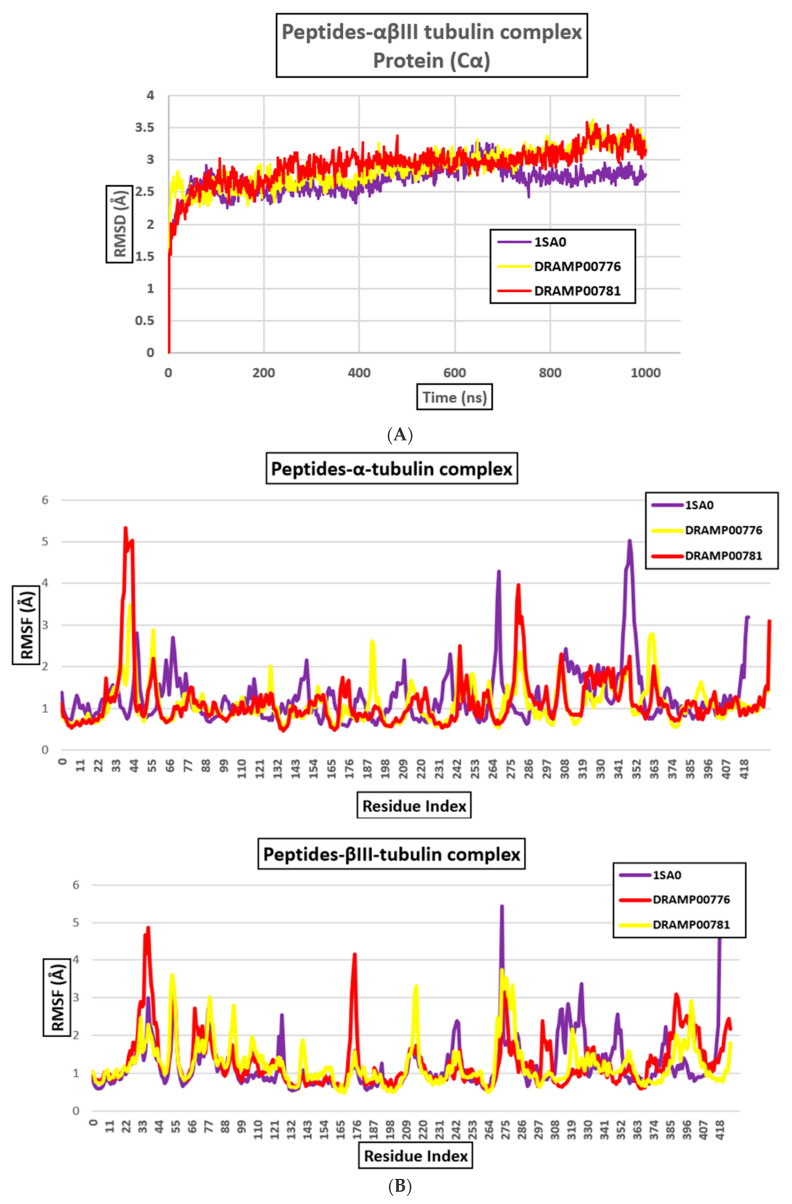
(**A**) RMSD for the αβIII-tubulin dimer after MD and (**B**) RMSF for the α and βIII-tubulin monomers.

The DRAMP00776 and DRAMP00781 peptides bind to the αβlll-tubulin dimer with binding energies of −43.38 and −32.08 kcal/mol, respectively, which represents a strong binding affinity of the peptides to the αβlll-tubulin dimer (Table 2 and Figure 7a). The docking result gives an idea about the principal binding sites of DRAMP00776 and DRAMP00781 on the α-tubulin monomer surface, to a lesser extent on the β-tubulin monomer surface. Figure 7b and Table 5 indicate that DRAMP00776 expressly interacts with αGlu22, αThr82, αArg229, αAsn18, αGlu22, αThr82, and αTyr224 on α-tubulin, and it also interacts with residues, βGly244, βLeu246, βPro243, on β-tubulin. While DRAMP00781 binds to residues αThr225, αArg229, αGlu22, αGlu22, αThr225, αGln11, αPro364, and αTyr224 on the α-tubulin surface, and residues βGly244, and βLeu246 on the β-tubulin surface. The αβ-tubulin interface exhibits hydrogen bonding with DRAMP00776 and DRAMP00781, within a 4 Å range. The docking results suggest that the hydrogen bonds are vital for binding between DRAMP00776 and DRAMP00781 and the αβlll-tubulin dimer. In contrast, hydrophobic and electrostatic interactions also provide a minimal contribution to stabilizing the complexes. To investigate the conformational fluctuations, the aggregate RMSD deviations of tubulin atomic coordinates were considered during the interaction of DRAMP00776 and DRAMP00781 with the αβlll-tubulin dimer (Figure 8A). A specific ligand should interact with the H6-H7 loop to efficiently inhibit the switch of nucleotide in tubulin (T216-Y224) [32,33]. Meanwhile, the H6-H7 loop favors hydrophobic interactions; thus, DRAMP00776 formed a hydrophobic interaction with αTyr224, while DRAMP00781 formed hydrophobic interactions with the βPro243 residue in the H6-H7 loop. Both peptides interact with residues at the H7-H8 loop (βGln245 and βGly244) via hydrogen bonding. As mentioned above, both peptides have a similar binding landscape and exhibit similar RMSD values of ~3.2 Å. The calculation of RMSF is an exceptional tool to determine local protein mobility. However, the rate of hydrogen bond formation may be directly implicated in the flexibility of peptides in the binding site, as shown in the RMSF graph (Figure 8B).

### 3.4. Peptide-αβIV-Tubulin Receptor Complex

**Figure 9:**
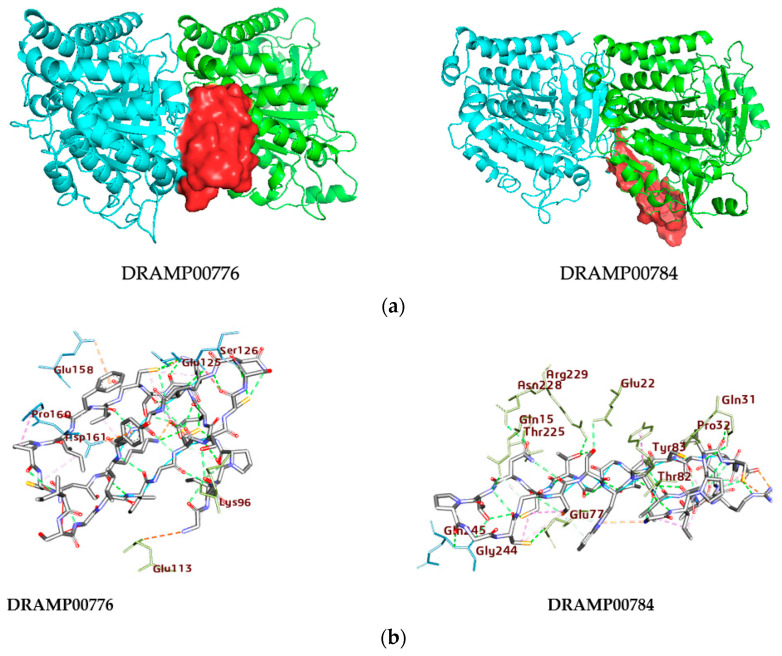
(**a**) The three-dimensional structures of DRAMP00776 and DRAMP00784 (red surface) occupy the binding site of the αβIV-tubulin dimer (light and flat red ribbon). (**b**) The two-dimensional structures of the DRAMP00776 and DRAM00784 αβIV-tubulin complexes show the range of the interactions and heterogeneity of the ligands’ positionings, as observed by docking. The proteins (green and light blue) are shown as sticks.

**Figure 10:**
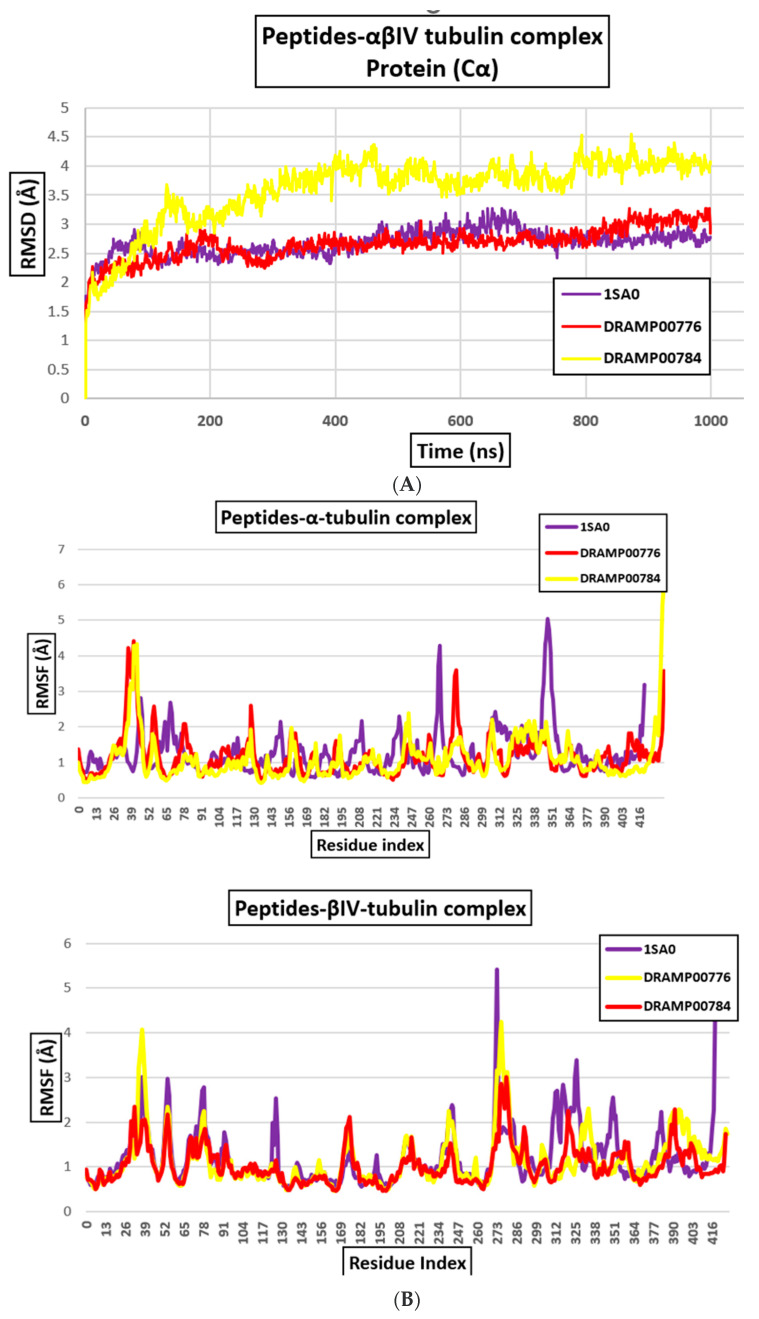
(**A**). RMSD of αβIV-tubulin dimer after MD simulations and (**B**) RMSF of α and βIV-tubulin protein monomers.

The DRAMP00776 and DRAMP00784 bind to the αβlV-tubulin receptor (Table 2 and Figure 9a) with binding energies of −42.27 and −48.97 kcal/mol, respectively, which shows a strong binding affinity of the peptides with the αβlV-tubulin receptor compared to their counterpart. The visualization of the docking result details the binding sites of DRAMP00784 and DRAMP00776 on αβIV-tubulin. DRAMP00776 and DRAMP00784 display a similar extent for the α and β-tubulin monomers. Figure 9b and Table 6 show that DRAMP00776 specifically interacts with αGlu113, and αLys96, at α-tubulin, and it also interacts with residues, βGlu158, βAsp161, βPro160, and βGlu125, in β-tubulin. By comparison, DRAMP00784 forms attractive charge interactions with residues αGln31, αThr82, αAsn228, αArg229, βGly244, and αGlu22, respectively. This is accompanied by four hydrogen bonds with αThr82, αThr225, αGln15, βGly244, and βGln245 (Figure 9b, Table 6). The αβ-tubulin interface exhibits hydrogen bonding with DRAMP00784 and DRAMP00776 within a 4 Å range. The stability of the DRAMP00784 and DRAMP00776 peptides with the proteins was observed for the complete length of the simulation. The RMSD analysis shows that the protein complexes made with DRAMP00784 and DRAMP00776 were stabilized after 70 ns of the simulation time, and this was maintained for DRAMP00776 until the completion of the simulation (Figure 10A). Meanwhile, the RMSD of DRAMP00784 displayed a deviation between 80 ns and 90 ns and continued in a stable conformation until the completion of the simulation. The two peptides maintained contact with the proteins by hydrogen bond interactions with residues αThr109, βPhe159, βPro160, αThr94, αGly95, βAsp128, βCys127 αGly95, βGlu125, and βAsp128 for DRAMP00776 and αGlu77, αAsn228, αGln15, αThr73, αAsn18, and βAsp41 for DRAMP00784. The RMSF values for each amino acid residue in the protein backbone are depicted in Figure 10B. The peaks represent the fluctuation of every amino acid residue throughout the simulation. This means that higher RMSF values represent greater flexibility of residues, while lower RMSF values reflect lower flexibility of residues and better system stability. The RMSFs of the α and β-domains are depicted separately. A slight amount of fluctuation in residues present at the active site indicates minimal conformational change, implying that the reported lead compound was consistently bound to the target protein.

### 3.5. Post-MM-GBSA Analysis

The post-MM-GBSA analysis of the free binding energy calculation was carried out by processing and analyzing the frame generated during molecular dynamics with a 10-step sampling size using the thermal MM-GBSA script in the Schrödinger suite. These are generally based on simulations of the molecular dynamics of the receptor–ligand complex. Consequently, they are intermediate in precision and computational effort between empirical scoring and strict alchemical disturbance methods [34]. These estimates have been applied to various systems with varying degrees of success. The free energies of binding have been enhanced after the post-MM-GBSA for all the analyzed compounds. The estimated values were negative for all the active docked complexes, of which DRAMP00776 showed the most negative MM/GBSA value (−109.02 kcal/mol), followed by DRAMP00783 (−87.04 kcal/mol) and DRAMP00789 (−64.01 kcal/mol) for the αβI-tubulin complex. DRAMP00789 peptides have low binding energy, which may have led to the instability of the peptides during the MD simulations, as represented in the RMSD and RMSF results. The results show that compound DRAMP00776 has a substantial binding energy based on the docked result and a significant post-MM-GBSA compared to the other compounds. These results indicate that the inhibition activity of DRAMP00776 against αβI-tubulin is likely to be more substantial compared to the other compounds. DRAMP00779 has a binding energy of −93.66 kcal/mol, whereas DRAMP00788 has a binding energy of −80.72 kcal/mol, which directly reveals the complex’s stability for αβII-tubulin. Notably, the high binding free energy was apparently due to strong electrostatic interactions, which demonstrate significant contributions of residues βLys324, αGlu77, αAsp76, βAsp355, and βAsp39, which were lacking in the binding of DRAMP00788. DRAMP00776 (−107.20 kcal/mol) exhibits superior predicted binding energy to the αβIII-tubulin dimer compared to the DRAMP00781 peptide (−106.12 kcal/mol). Moreover, both peptides show good stability results in this study, which corroborate with their ClusPro and the global energy value obtained in the docking analysis. Nevertheless, this provides initial evidence that these peptides represent potential candidates for the development of novel therapeutic applications to cancer cell inhibition, which renders them suitable for further examination. The binding energy calculation of the peptides-αβIV-tubulin complex shows that the average binding free energies of the DRAMP00776 and DRAMP00784 docked complexes are −102.97 and −104.90 kcal/mol, respectively. The resulting binding energy difference could be due to hydrophobic interactions between DRAMP00784 and the αTyr224 residue (H6-H7 loop), which are lacking in the DRAMP00776 docked complex. The MM-GBSA data suggest that the intermolecular electrostatic, hydrophobic, and hydrogen bonding interactions are vital in the binding of the peptides to the respective tubulin receptor sites. The more pronounced stabilization observed in the evaluated peptides appears to correlate with their binding energy. Importantly, the activity or inactivity of the peptides that inhibit tubulin polymerization corresponds to their tubulin-binding ability as assessed by the molecular dynamics simulation results [11].

### 3.6. Physicochemical, Allergenicity, and Toxicity Prediction

The physicochemical properties of the peptide sequences of interest were generated using the Expasy web server tool. The following properties were examined: length, aliphatic index, instability, and molecular weight (Table 7). Peptides DRAMP00782 and DRAMP00789 were determined to be unstable, with an instability index of 50.50 and 50.46, respectively. These peptides will most likely also be found unstable in vitro because a value of the instability index above 40 is considered unstable. Thus, preventive procedures are to be taken to stabilize the unstable therapeutic peptides through suitable biochemical processes. In comparison, the remaining peptides are predicted to be stable. Further, the sizes of the peptides range from 378 to 434 atoms, with molecular weights between 2902 and 3199 Dalton. The theoretical isoelectric point (pI) denotes the respective pH of the peptides. The predicted aliphatic indices of the peptides were found to be 84.84, 46.90, 74.67, and 50.35 for peptides DRAMP00776, DRAMP00782, DRAMP00783, and DRAMP00789, respectively. The aliphatic index often indicates the relative volume of the aliphatic lateral chains (alanine, valine, isoleucine, and leucine). This is a positive factor in increasing the thermostability of globular proteins. In terms of residue charge, DRAMP00776, DRAMP00779, and DRAMP00783 tended to be charged negatively. AllerTOP was used for the in silico prediction of allergens based on the primary physicochemical properties of proteins. Meanwhile, the application uses the amino acid z-descriptors, ACC protein transformation, and k nearest neighbors clustering parameters. Most of the peptides are non-allergic and non-toxic, apart from the DRAMP00779 and DRAMP00783 peptides, which are identified as possible allergens. The ToxinPred results for all the active peptides showed that they were non-toxic compared to the mutated peptides.

Interestingly, all the peptides belong to the cyclotide family, with most of them classified as plant defensins. Cyclotides are macrocyclic peptides with a knotted arrangement of three disulfide bonds formed from their six conserved cysteine residues. They contribute to their exceptional stability and natural functions as plant defense peptides. Cyclotides have many pharmaceutically relevant activities, especially their significance in drug design. Several synthetic cyclotides have also been made for applications in drug design. The first cyclotides were generated during the rise of natural products through their discovery as active compounds in studies that screened plant extracts for medicinal properties [35,36,37,38]. They were initially discovered because of their uterotonic activity by identifying Kalata B1 as an active agent from *Oldenlandia affinis*, thus used in African traditional medicine as a utertonic tea to quicken childbirth [39]. Circulin A and circulin B isolated from *Chassalia parvifolia* extract have been reported to act as anti-HIV agents [40]. Hence, further explorations of the best plant-based peptides analyzed in this work, as tubulin polymerization agents are hoped to be of great benefit. After the MD analysis, all the peptides were found to lack interactions with residue β45. This may affect the microtubule dynamics in the class βII-VIII isotypes [3]. Our results also conclude that the different β tubulin isotype interactions with the peptides are unique. Among the docked peptides, DRAMP00776 was found to be involved in strong interactions with αβI, αβIII, and αbIV tubulin proteins. However, it shows superior binding energy with αβI (−109.02 kcal/mol) and αβIII (−107.20 kcal/mol), compared to αβIV (−102.97 kcal/mol). Numerous preclinical studies have demonstrated that elevated levels of βIII-tubulin expression are linked with drug resistance in human cancer cell lines such as lung, ovary, prostate, and cancer [41]. On the other hand, the βI-tubulin isotype is the most highly expressed tubulin isoform in humans and the most common isotype found in cancerous cells, hence its importance as a target for inhibition in cancer cells. Chemical synthesis can readily produce the promising peptide for the respective tubulin isotypes since specific differences will result in an improved therapy protocol. Moreover, recombinant technologies can be employed to satisfy the multiple prerequisites, which are in place in the pharmaceutical sector. Peptides can be efficiently designed, functionalized, and modified to optimize their bioavailability, stability, specificity, and effectiveness to enable the peptide to fulfill clinical drug requirements [42,43].

## 4. Conclusions

An in silico analysis of the protein–peptide interactions by docking using tubulin dimers as targets was carried out in sequence with molecular dynamics. In this study, the four most important isotypes of β-tubulin were explored (βI–βIV). Reported peptides with potential anticancer properties can serve as potential candidates for developing new therapeutic options for destabilizing microtubules. Consequently, they can provide candidate structures for anticancer applications. Interfering with mitotic spindle formation is a time-tested strategy for cancer chemotherapy design and development. The molecular dynamics simulation was further explored to rule out false interactions and investigate the stability of the proteins when interacting with the selected peptides. In addition, allergenicity profiling confirmed the non-allergic properties of the peptide molecules selected in this current study. The subsequent data analysis has led to the identification of the most promising peptide molecules that bind to the residues in the tubulin dimer and thus inhibit its polymerization dynamics. The presence of minor variations of peptide binding in the protein structure of β-tubulin isoforms can be an initial step for developing a new medicinal product with high levels of selectivity and specificity for a tubulin isotype of choice. This can ultimately lead to the development of secondary lines of treatment for cancer cell types that are pharmaco-resistant after conventional chemotherapy fails due to somatic mutations, changed expression levels of target proteins, or increased expression of efflux pumps. We hope that this research will provide further insights and possibly a new focus for the future development of drugs and/or biologics that target critical regions within tubulin. We have aimed here to initiate investigations of a new generation of potential peptide-based therapeutics that would selectively and specifically target tubulin variants and thus help reduce the adverse side effects associated with a vast majority of the present cancer chemotherapy drugs.

## Figures and Tables

**Table 1:** The peptide IDs, amino acid sequences, and the sequence lengths/alignment curated from the data repository of antimicrobial peptides database are listed.

DRAMP_ID	Sequence	Sequence Length
DRAMP00770	GEYCGESCYLIPCFTPGCYCVSRQCVNKN	29
DRAMP00775	GIPCGESCVFIPCLTSAIDCSCKSKVCYRN	30
DRAMP00776	GSIPCEGSCVFIPCISAIIGCSCSNKVCYKN	31
DRAMP00777	GSIPCGESCVFIPCISAIIGCSCSSKVCYKN	31
DRAMP00778	GSIPCGESCVFIPCISAVIGCSCSNKVCYKN	31
DRAMP00779	GIPCGESCVFIPCISSVIGCSCSSKVCYRN	30
DRAMP00780	GSIPCGESCVFIPCISSVIGCACKSKVCYKN	31
DRAMP00781	GLPVCGETCVGGTCNTPGCGCSWPVCTRN	29
DRAMP00782	GLPVCGETCVGGTCNTPGCACSWPVCTRN	29
DRAMP00783	GIPCGESCVWIPCITSAIGCSCKSKVCYRN	30
DRAMP00784	GLPVCGETCVGGTCNTPGCSCSWPVCTRN	29
DRAMP00788	GLPICGETCVGGTCNTPGCSCSWPVCTRN	29
DRAMP00789	GVPICGETCTLGTCYTAGCSCSWPVCTRN	29

**Table 2:** Protein–peptides docking information resulting from PATCHDOCK (refined with FIREDOCK) and ClusPro score.

Peptide ID	Global Energy (kcal/mol)	Attractive eVdW	Repulsive eVdW	ACE	HB	ClusPro Score
Peptide—αβI-tubulin receptor complex						
DRAMP00776	−65.14	−32.53	16.98	−10.96	−1.90	−738.00
DRAMP00782	−65.44	−33.64	14.21	−8.05	−3.08	−858.20
DRAMP00783	−56.63	−38.80	20.39	−7.62	−7.55	−761.90
DRAMP00789	−54.12	−29.92	−19.61	−13.64	−2.00	−983.30
Peptide—αβII-tubulin receptor complex						
DRAMP00779	−63.91	−35.63	24.48	−4.19	−3.61	−747.00
DRAMP00788	−52.51	−37.06	25.00	−10.52	−3.41	−942.20
Peptide—αβIII-tubulin receptor complex						
DRAMP00776	−43.38	−29.11	10.37	−4.88	−3.59	−736.40
DRAMP00781	−32.08	−22.85	10.24	−6.45	−1.65	−735.60
Peptide—αβIV-tubulin receptor complex						
DRAMP00776	−46.27	−24.00	11.67	−6.06	−2.46	−743.40
DRAMP00784	−48.97	−42.22	45.90	−6.11	−8.23	−829.13

VdW: van der Waals interaction; ACE: atomic contact energy (desolvation energy); HB: hydrogen bond.

**Table 3:** Interaction types in the peptide–αβI-tubulin complex identified before and after molecular dynamics simulations.

Before MD			After MD		
Residues	Distances (Å)	Interaction Types	Residues	Distances (Å)	Interaction Types
**DRAMP00776**					
αAsp224	4.67	Attractive charge	αAsp224	2.79	Salt bridge
αThr221, αArg359, βArg46, αGly223, βAsp355	2.17, 3.10, 3.04, 3.68, 3.63,	Conv–H and C–H bonds	αLys19, αAsn226, αArg276, αGln15	2.67, 3.29, 2.73, 3.00, 2.70	Conv–H bond
αHis227	3.30	Pi–sigma	αGly71, αSer75, αGly79, αPro80, αGly223, αGlu22	3.46, 3.60, 3.15, 3.59, 3.45, 3.54, 3.80	C–H bond
			αLys19	3.89	Pi–cation; Pi–donor Hydrogen bond
			αLeu215, αArg276, βMet323	5.37, 4.61, 5.28	Alkyl
			αHis227, αVal76, αLys19	4.87, 5.19, 4.86	Pi-alkyl
**DRAMP00782**					
αLys19	2.53457	Salt bridge	αLys19	1.53	Salt bridge; attractive charge
βAsp41, βGlu45, αGlu22	4.52, 4.46, 4.99	Attractive charge	βAsp41	2.78	Attractive charge
βAsp355, αLys19, αGly223	2.71, 3.35, 2.40	Conv-H and C-H bonds	αGln15, αGly223, αSer25	1.67, 1.97, 1.81	Conv–H bond
βPro243, βLeu42	4.54, 4.94	Alkyl	αLys19, αAsp31, αPro32, αThr221, αGly223 αGlu22	2.41, 2.77, 2.75, 2.83, 2.89, 2.43	C–H bond
			βLeu42, αPro80	4.87, 4.75	Alkyl
**DRAMP00783**					
αSer78, αGly79, αTyr222, αGly223, αAsp224, βAsp355, αGln15, αSer75 αThr221, αSer78 αAsp224	2.89, 3.47, 2.18, 2.54, 3.21, 2.62, 3.15, 3.14, 2.32, 2.49, 3.11	Conv–H and C–H bonds	αAsp224 βAsp41	1.77, 1.72, 1.58	Salt bridge; attractive charge
βLeu42	4.61255	Alkyl	αAsp74, βAsp41, αAsp177	4.29, 5.25, 4.92,	Attractive charge
			αGln15, βAsp355, βGln245	2.39, 1.92, 1.68, 1.77, 2.48, 1.77, 1.69, 2.54, 1.93, 1.86	Conv–H bond
			αAsp74 βAsp355	2.86, 2.65, 2.84, 2.87	C–H bond
			βMet323, βVal353	5.20, 5.19	Alkyl
			αTyr222	4.91	Pi–alkyl
**DRAMP00789**					
αGln279, βMet321, βMet323, αThr219, βAsp355, βGln245, αGly360, βSer322, βASP355	2.45, 3.03, 3.09, 2.90, 2.40, 3.13, 3.29, 3.25, 3.04, 3.41,	Conv-H and C–H bonds	αAsp26	2.70, 4.90	Salt bridge/attractive charge
βArg320	5.14	Alkyl	αLys19, βMet323, αThr219, αAsp26, αArg282, αGly360	3.29, 3.25, 2.79, 2.63, 3.18, 3.05	Conv–H bond
			αPro32, αSer275, αSer78, αSer78, αGly79	3.42, 3.78, 3.78, 3.45, 3.38	C–H bond
			αPro32, αPro80, αArg359	4.88, 4.59, 4.87	Alkyl

**Table 4:** Interaction types in the peptide–αβII-tubulin complex described before and after molecular dynamics simulations.

Before MD			After MD		
Residues	Distances (Å)	Interaction Types	Residues	Distances (Å)	Interaction Types
**DRAMP00779**					
βGlu45	2.61	Salt bridge	βLys324, αAsp76, αGlu77, βAsp355	2.54, 2.65, 2.72, 2.7	Salt bridge
αArg221, αGlu279, βGlu45, αGlu77	4.35, 5.4, 5.08, 4.14	Attractive charge	βAsp39, βAsp355	5.10, 4.87	Attractive charge
αThr82 βGly244	2.41, 2.51, 3.00	Conv–H bond	αGly81, αArg221, βMet323, αGly81, αThr80, βAsp355	2.95, 3.00, 3.71, 2.89, 2.83, 2.81	Conv–H bond
αThr225, βGln245, αThr82, αGly365	3.32, 2.442, 3.06, 2.38	C–H bond			
αArg229	3.32	Pi–cation	αArg221, αGly365, βMet321	3.52, 3.79, 3.46	C–H bond
αTyr83, αAla19, αArg229	3.63, 4.03, 4.94	Pi–alkyl	βPro243, βMet323, βLeu42	5.06, 5.20, 4.61, 5.31	Alkyl
**DRAMP00788**					
βAsp41	4.44	Attractive charge	αGlu279, αAsp218	1.63, 1.51	Salt bridge; attractive Charge
αArg221	2.90	Conv–H bond	βAsn247, βVal353, βAsp355, αThr73, βVal353, αGlu22, αThr82	2.01, 2.74, 2.47, 2.50, 2.52, 1.64, 1.98	Conv–H bond
βLeu246, βAsp41	2.41, 3.42	C–H bond C–H bond	αThr82, Ser178, αSer178, αPro364, βGly244, βGln245, βAla352, αLeu217, βleu246, αVal177, βGln245	2.53, 2.71, 3.09, 2.27, 2.75, 2.79, 2.41, 3.02, 2.40, 2.50, 2.57, 2.63	C–H bond
αThr225	3.44, 3.30	Pi–donor	αThr225	2.65	Pi–donor hydrogen bond
αTyr224	5.23, 3.85	Pi–alkyl	αIle219, αLeu217, αPro364, αPro364	5.18, 5.14, 4.63, 4.92	Alkyl

**Table 5:** Interaction types in the peptide–αβIII-tubulin complex depicted before and after molecular dynamics simulations.

Residues	Distances (Å)	Interaction Types	Residues	Distances (Å)	Interaction Types
**DRAMP00776**					
αGlu22	4.63	Attractive charge	αGlu22	4.67	Attractive charge
αThr82, αArg229, βGly244, βLeu246	3.22, 2.37, 2.76, 3.16	Conv–H bond	αGln11, αThr82, βGln245 βGly244, αGlu77, αGln15, αGln15, αGly366	1.91, 2.60, 2.17, 1.98, 1.89, 3.09, 2.87, 2.10, 2.53, 2.08	Conv–H bond
αAsn18, αGlu22, αThr82	3.21, 2.37, 3.28	C–H bond	αGly81, αThr225, αArg229, βGly244, αGln15, αGly365, αGly366	2.68, 2.56, 2.36, 2.65, 2.53, 2.74, 2.88, 2.61	C–H bond
βPro243	5.10	Alkyl	βMet323, αVal74	4.81, 5.12	Alkyl
αTyr224	4.76	Pi–alkyl	αTyr224	4.41	Pi–alkyl
**DRAMP00781**					
αThr225, αArg229, αGlu22	2.32, 2.59, 2.79, 3.08	Conv–H bond	αGln15, αTyr83, βArg46, βGly244, αThr82, αGlu22, αGln15, αVal74, αThr73, αGlu77, αGly29, αIle30	1.93, 1.81, 2.94, 1.89, 2.71, 2.50, 1.81, 1.67, 2.93, 2.89, 1.89, 1.91, 2.81, 2.67	Conv–H bond
αGlu22, αThr225, αGln11, βGly244, βLeu246	2.32, 2.88, 3.62, 2.27, 3.56	C–H bond	αPro32, αThr82, αGlu22, αThr73, βGln245, αGlu77	2.79, 2.73, 2.76, 2.68, 2.92, 2.98	C–H bond
αPro364	3.73, 4.39	Alkyl	αVal363, αPro364, αPro364, αPro32, αLeu26, βLeu42, βPro243	4.51, 4.70, 5.08, 5.30, 4.69, 4.71, 4.76	Alkyl
αTyr224	3.83	Pi–alkyl			

**Table 6:** Interaction types in the peptide-αβIV-tubulin complex listed before and after molecular dynamics simulations.

Residues	Distances (Å)	Interaction Types	Residues	Distances (Å)	Interaction Types
**DRAMP00776**					
αGlu113, βGlu125	5.19, 4.55	Attractive charge	βGlu125	1.73	Salt bridge; attractive charge
βGlu125	2.44, 3.02	Conv–H bond	αGly95, βGlu125, βAsp128	2.32, 2.83, 3.09	Conv–H bond
αLys96, βSer126	3.20, 2.32, 3.02	C–H bond	αThr109, βPhe159, βPro160, αThr94, αGly95, βAsp128, βCys127	2.95, 2.65, 2.45, 2.56, 2.57, 2.81, 2.74, 2.95, 2.45, 2.38	C–H bond
βGlu158, βAsp161	4.49, 4.20	Pi–anion	βArg162	4.73,	Pi–cation
**DRAMP00784**					
αGln31, αThr82, αAsn228, αArg229, βGly244, αGlu22	2.00, 2.30, 2.88, 1.88, 2.45, 2.65, 2.61, 1.93, 2.34	Conv–H bond	αAsp76	4.89	Attractive charge
αThr82, αThr225, αGln15, βGly244, βGln245	2.47, 2.83, 2.68, 2.48, 2.65, 2.83, 2.85, 2.53, 2.58, 2.54	C–H bond	αGlu77, αAsn228, αGln15, αThr73, αAsn18, βAsp41	3.54, 3.31, 2.81, 3.00, 3.04, 3.00	Conv–H bond
αPro32	4.97	Alkyl	βLeu246	5.07	Alkyl
αTyr83	5.11	Pi–alkyl	αTyr224	4.44	Pi–alkyl

**Table 7:** The physicochemical, allergic, and toxicity profile of the best-docked peptides.

	Physicochemical Properties							Toxicity	Allergenicity
Peptide ID	Aliphatic Index	Instability Index	Theoretical pl	Total Number of Negatively Charge Residues (Asp + Glu)	Total Number of Negatively Charge Residues (Asp + Glu)	Total Number of Atoms	Mol wt	Prediction	AllerTOP v. 2.0
DRAMP00776	84.84	26.45	7.77	1	2	437	3197.24	Non-toxin	Probable non-allergen
DRAMP00779	81.00	31.50	7.77	1	2	423	3113.1	Non-toxin	Probable allergen
DRAMP00781	0.162	35.35	5.92	1	1	378	2872.71	Non-toxin	Probable non-allergen
DRAMP00782	46.90	50.50	5.96	1	1	381	2886.73	Non-toxin	Probable non-allergen
DRAMP00783	74.67	18.66	8.33	1	3	434	3179.21	Non-toxin	Probable allergen
DRAMP00784	43.45	46.59	5.96	1	1	382	2902.32	Non-toxin	Probable non-allergen
DRAMP00788	46.90	39.95	5.96	1	1	385	2916.76	Non-toxin	Probable non-allergen
DRAMP00789	50.34	52.46	5.96	1	1	395	2983.84	Non-toxin	Probable non-allergen

Instability index of peptide <40—stable, and >40—unstable; Mol wt: molecular weight.

## Data Availability

The final dataset used for this manuscript can be requested from the corresponding author.

## Supplementary Materials

The following supporting information can be downloaded at: https://www.mdpi.com/article/10.3390/jfb14030135/s1, Table S1. Residues (%) in the different regions of human αβ-tubulin isotypes using PROCHECK; Figure S1. Ramachandran plot of human αβI-αβIV tubulin isotypes from PROCHECK.
