# Supplementary material for: A Molecular Docking Study Reveals That Short Peptides Induce Conformational Changes in the Structure of Human Tubulin Isotypes αβI, αβII, αβIII and αβIV
Source: J Funct Biomater. 2023 Feb 28;14(3):135. doi: 10.3390/jfb14030135 (PMC10054586; doi:10.3390/jfb14030135)
Supplement: Supplementary file 1 [file jfb-14-00135-s001.zip › jfb-2216066-supplimentary.pdf]

A Molecular Docking Study Reveals That Short Peptides Induce Conformational Changes in the Structure of Human Tubulin Isotypes αβI, αβII, αβIII and αβIV

Oluwakemi Ebenezer [1,4], Nkululeko Damoyi [1], Michael Shapi [1], Gane Ka-Shu Wong [2,3] and Jack A. Tuszynski [3,4,5,6,*]

[1] Department of Chemistry, Faculty of Natural Science, Mangosuthu University of Technology, Umlazi 4031, South Africa
[2] Department of Biological Sciences, University of Alberta, Edmonton, AB T6G 2E9, Canada
[3] Li Ka Shing Institute of Virology, University of Alberta, Edmonton, AB T6G 2E1, Canada
[4] Department of Physics, University of Alberta, Edmonton, AB T6G 2E1, Canada
[5] Department of Oncology, Cross Cancer Institute, University of Alberta, Edmonton, AB T6G 1Z2, Canada
[6] Department of Mechanical and Aerospace Engineering, (DIMEAS), Politecnico di Torino, 10129 Turin, Italy
* Correspondence: jackt@ualberta.ca

Table S1. Residues (%) in the different regions of human αβ-tubulin isotypes using PROCHECK

αβ-Tubulin isotypes	Residues in the most favored regions (%)	Residues additional allowed regions (%)	Residues in the generously allowed regions (%)	Residues in the disallowed regions (%)
αβI	85.1	11.4	2.3	1.2
αβIIa	83.4	13.3	2.3	1.1
αβIII	83.7	12.9	2.4	1.1
αβIVa	84.1	12.7	2.3	0.9

PROCHECK

Ramachandran Plot

αβI isotype

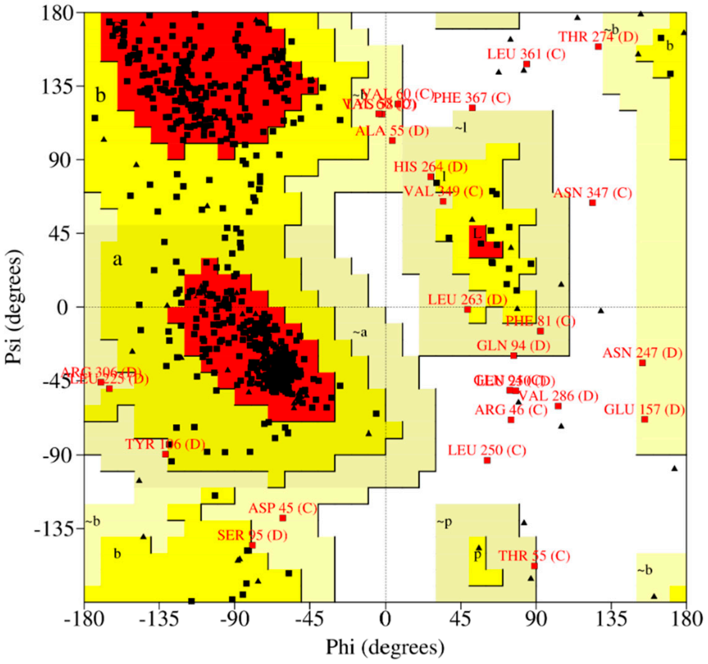

Plot statistics

Residues in most favoured regions [A,B,L]	629	85.1%
Residues in additional allowed regions [a,b,l,p]	84	11.4%
Residues in generously allowed regions [~a,~b,~l,~p]	17	2.3%
Residues in disallowed regions	9	1.2%
	----	------
Number of non-glycine and non-proline residues	739	100.0%
Number of end-residues (excl. Gly and Pro)	4	
Number of glycine residues (shown as triangles)	67	
Number of proline residues	40	

Total number of residues	850	

Based on an analysis of 118 structures of resolution of at least 2.0 Angstroms and R-factor no greater than 20%, a good quality model would be expected to have over 90% in the most favoured regions.

A

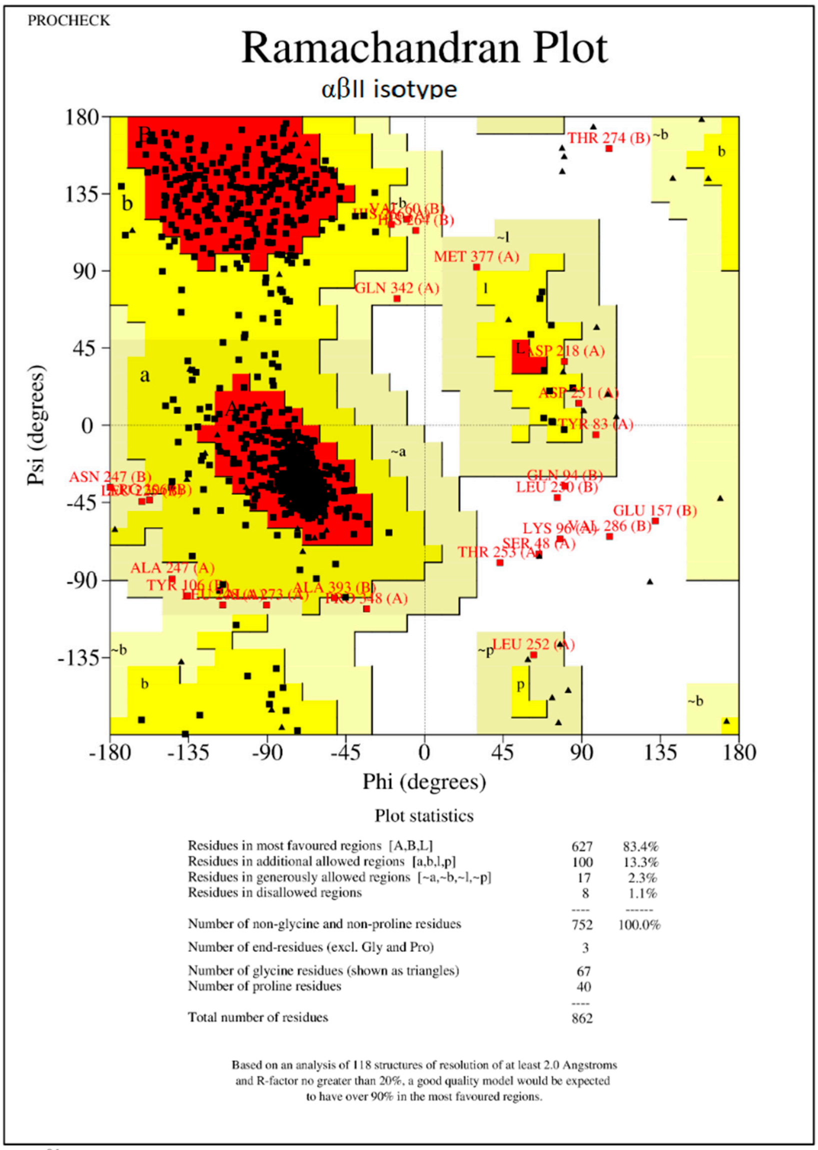
PROCHECK
Ramachandran Plot
αβII isotype
Psi (degrees)
Phi (degrees)
Plot statistics
Residues in most favoured regions [A,B,L] 627 83.4%
Residues in additional allowed regions [a,b,l,p] 100 13.3%
Residues in generously allowed regions [~a,~b,~l,~p] 17 2.3%
Residues in disallowed regions 8 1.1%
Number of non-glycine and non-proline residues 752 100.0%
Number of end-residues (excl. Gly and Pro) 3
Number of glycine residues (shown as triangles) 67
Number of proline residues 40
Total number of residues 862
Based on an analysis of 118 structures of resolution of at least 2.0 Angstroms and R-factor no greater than 20%, a good quality model would be expected to have over 90% in the most favoured regions.

saves_01.ps

B

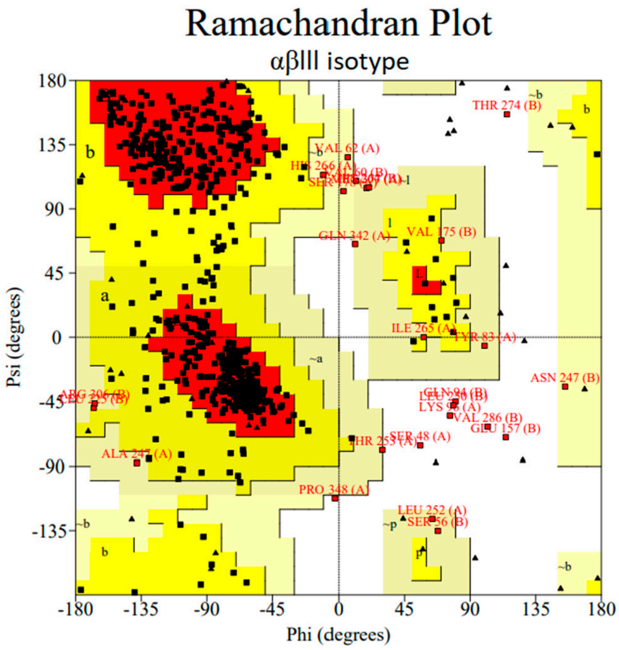

Plot statistics

Residues in most favoured regions [A,B,L]	634	84.2%
Residues in additional allowed regions [a,b,l,p]	95	12.6%
Residues in generously allowed regions [~a,~b,~l,~p]	17	2.3%
Residues in disallowed regions	7	0.9%
	----	------
Number of non-glycine and non-proline residues	753	100.0%
Number of end-residues (excl. Gly and Pro)	6	
Number of glycine residues (shown as triangles)	66	
Number of proline residues	39	

Total number of residues	864	

Based on an analysis of 118 structures of resolution of at least 2.0 Angstroms and R-factor no greater than 20%, a good quality model would be expected to have over 90% in the most favoured regions.

C

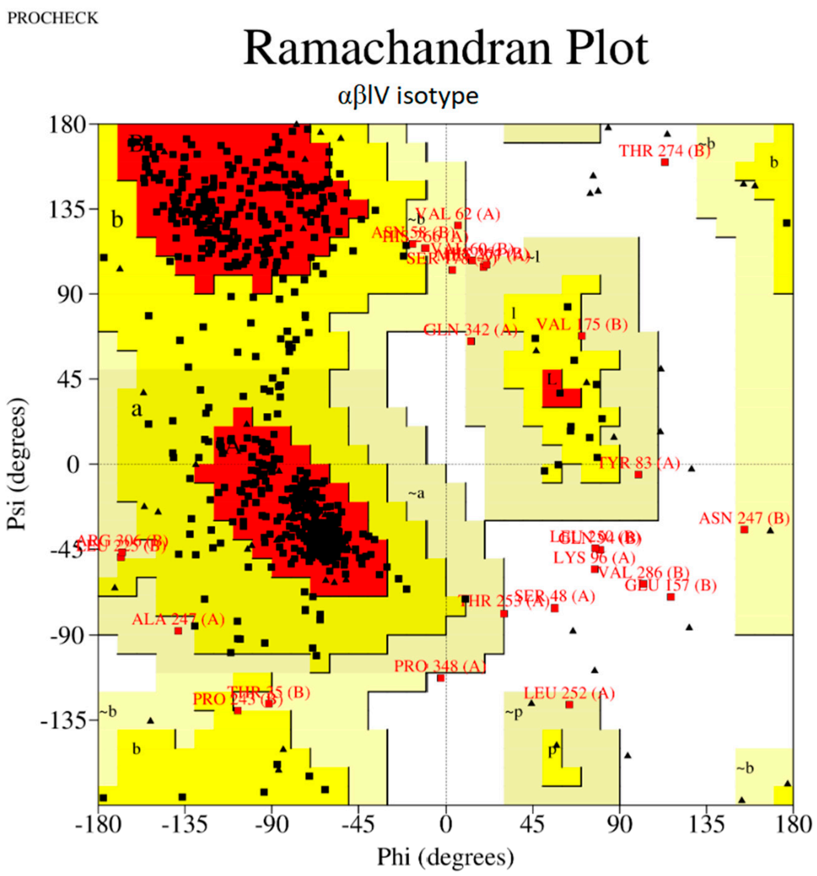

Plot statistics

Residues in most favoured regions [A,B,L]	631	84.1%
Residues in additional allowed regions [a,b,l,p]	95	12.7%
Residues in generously allowed regions [~a,~b,~l,~p]	17	2.3%
Residues in disallowed regions	7	0.9%
	----	------
Number of non-glycine and non-proline residues	750	100.0%
Number of end-residues (excl. Gly and Pro)	7	
Number of glycine residues (shown as triangles)	68	
Number of proline residues	40	

Total number of residues	865	

Based on an analysis of 118 structures of resolution of at least 2.0 Angstroms and R-factor no greater than 20%, a good quality model would be expected to have over 90% in the most favoured regions.

D

Figure S1. Ramachandran plot of human αβI-αβIV tubulin isotypes from PROCHECK
